# Online Academics in Pakistan: COVID-19 and Beyond

**DOI:** 10.12669/pjms.37.1.2894

**Published:** 2021

**Authors:** Nazia Mumtaz, Ghulam Saqulain, Nadir Mumtaz

**Affiliations:** 1 Dr. Nazia Mumtaz, PhD. (Rehabilitation Sciences) Head of Department of Speech Language Pathology, Faculty of Rehab & Allied Health Sciences, Riphah International University, Islamabad, Pakistan; 2 Dr. Ghulam Saqulain, F.C.P.S (Otorhinolaryngology) Head of Department of Otolaryngology Department of ENT, Capital Hospital, Islamabad, Pakistan; 3 Mr. Nadir Mumtaz, LLB Former DG Research, FBR Islamabad, Pakistan

**Keywords:** COVID-19, Coronavirus, SARS-CoV-2, Education, Online Academics

## Abstract

COVID-19 is a menace for Pakistan’s fragile and overburdened health care system and infrastructure, insidiously permeating the socio-economic fabric. Globally complete to partial shutdown of educational institutions has been enforced, transitioning from face to face to online academics. Academic institutes are floundering to withstand the brunt. Therefore, the current study was conducted to attempt to review and highlight the impact and challenges posed by transition from conventional to online academics and how to approach them, in the wake of COVID-19 pandemic with the perspective of developing countries like Pakistan. For this purpose, search for relevant literature using search engines and websites including Google, Google Scholar and Web of Science as well as Medline database was conducted with keywords “Covid-19, academics, mental health, social impact and e-learning and combination of words”. Thirty two English language, full text articles published in the last ten years from 2010 to 2020 were selected for the literature review. With this literature review, we conclude that this lockdown has caused significant distortion in the academic world yet unequal interruption in learning with significant disruptions in internal assessments and qualification examinations with developing countries like Pakistan, compounded by a compromised educational system. However, COVID-19 is spurring the case for conversion to online academics and developing countries like Pakistan are poised to develop reliable, cost effective and secure online academic system whether it is bane or boon.

## INTRODUCTION

The term “Global village” has now become synonymous with the term “Global Pandemic” recognizing no geographical border, affecting the affluent Western countries more in terms of mortality. Africa and swathes of South Asia and Far East apparently have escaped the brunt.^1^ The West has the financial strength to weather out the pandemic, yet such technological advancement is not available to the developing world. A common concern is the academic vista of those enrolled in educational institutions in Pakistan and the scurrying of the Higher Education Commission (HEC) and universities to convert to online academic and a deviation from conventional instructional methodology. The underlying sentiment is that COVID-19 may persist until the virus runs its life cycle in the backdrop of any scientific curative breakthrough.

This strain of virus (Novel corona or SARS-CoV-2) has been declared as a global emergency by World Health Organization.^2^ In Pakistan it is not clear if co-morbidities are the underlying factor for COVID-19 related fatalities or such fatalities are being attributed towards the virus. The vaccine for SARS-CoV-2 has to pass the clinical trial stages. Travel restrictions have seriously impacted academicals routines.^3^ The West remains engrossed in their response to the threat of COVID-19 and reconciled to an educational shutdown, including losing precious academic semesters at all levels with more than 100 nations enforcing closure of educational institutions.^4^ In Pakistan students and faculty remain confined to homes yet maximum utilization of time can be productively made across educational disciplines including research. Private sector universities have oriented towards digital academics, yet the federal education authorities seem to have abdicated from their moral authority by citing constitutional provisions under the cover of 18^th^ amendment of the constitution of Pakistan. An underlying reason for this advertent neglect is that the teachers’ jobs in the federation and provinces are protected as being paid from the taxpayers’ money.

A transition from conventional teaching to online delivery of programs and courses is underway.^5^ With conventional education system being a skill development tool, capable of raising skills and social awareness, institutions stopped in-person teaching and are opting for virtual settings to promote learning. In Pakistan HEC is committed to make e-learning a success in the wake of COVID-19 pandemic, with only some previous efforts made by Open University,^6^ while some universities already commenced online classes and stragglers take the refuge of summer break to prepare for transition to online academics.

With around 90% of world population affected by such closures,^3^ UNESCO shared recommendations for uninterrupted online learning for the lockdown period, however detractors opine that online teaching is complicated due to the poor economic conditions in the middle income countries while losing sight of the fact that application like Facebook are widespread in Pakistan.

**Fig.1:**
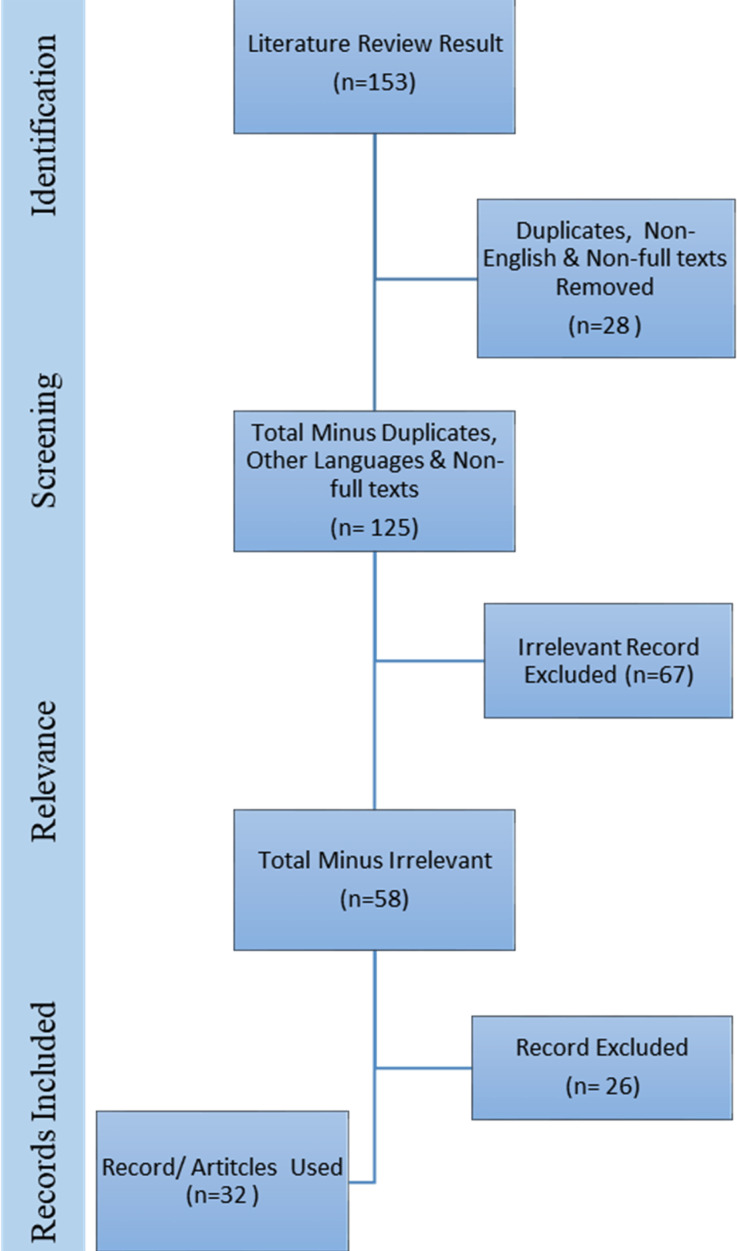
Diagrammatic representation of search strategy.

A significant by product of online academics is cost reduction in terms of physical infrastructure. Virtual universities in some developing countries are confronted with the paradigm of synching and integrating of online into research factored distant education as the next academic frontier.^7^ Hence, this paper attempts to review challenges posed by transition to online academics and how to approach them in the wake of current pandemic with perspective of developing countries like Pakistan. For this purpose, to broaden the search for relevant literature search engines and websites including Google, Google Scholar and Web of Science as well as Medline database search was conducted. The keywords used for the search including “COVID-19, academics, mental health, social impact and e-learning. The Boolean operator “And” and “Or” were also used employing the said keywords. The search was limited to the articles published in the last ten years from 2010 to 2020. Duplicates, articles in other languages, and articles in which full-text was not available were removed. Initially skimming technique was used to select articles on titles of relevance, which resulted in 58 articles, of which 26 were excluded following the review and 32 articles were selected for the literature review (Fig.1).

## DISCUSSION

Compared to western countries like United States of America where institutions offered 89% courses online and out of those more than 50% offered full degree programs completely online,^8^ in the developing countries the picture was dismal with only some open universities partly offering such programs before the COVID-19 lockdowns.^6^ The challenges posed by transition from conventional education system to online academics and how to approach them in the wake of COVID-19 threat in developing countries like Pakistan is discussed.

### Education

With the threat of spread of virus, academic institutes are confronted with the looming specter of digital infrastructure, bandwidth and licensed software applications. Hence, an invigorated HEC, initiated inculcation and training of faculty and staff in the realm of online modalities in the entire spectrum of undergraduate to doctoral level through an enabling environment. This concept is strengthened by a previous study conducted by Badasyan and Silva which reported significant improvement in test scores in native language and math’s tests.^9^ Also in an Eastern Mediterranean study involving English preparatory students, majority revealed that they can use internet as an educational tool,^10^ if accompanied by resilience.^11^ The major issues expected to arise are in the disciplines requiring clinical training which require hands as well as interactive environment and although not as effective through a digital interface artificial cadavers is a moot point as a practice already in place. As reflected in an Indian study involving dental students to adopt digital revolution in education^12^ hence, the proverbial bridge can be crossed. The Radiology department of local tertiary care center has started tasking residents with radiological images delivered online for quiz.^13^ Since, clinical education has been severely affected development of education consortiums for residents may be required.^14^ In countries with advanced healthcare systems, telehealth practices are in vogue covering the clinical component aspect.

The sudden immersion into online academics and unreliable attendance pattern and dissimilar video online applications need to be worked upon and developed in harmony to dispel any paradox. The online situation into which the academics both teachers and students have been thrust is, unforeseen with students perceiving that readiness was lacking among staff and institution.^11^ In this backdrop HEC rising out of its slumber and perhaps as a reaction to the initiatives of one University even convened a meeting of Vice Chancellors.

### Accessibility

In the developing countries, access to technology may not be affordable to all and 80% students may remain deprived of computers and internet and the need for cheap, rugged equipment for the rural students in the developing countries persists.^15^ In a Nigerian study by Jibrin MA et al. reported that challenges faced in the use of internet were its speed as well as deficient stable power supply and recommended support by government for tertiary institutions.^16^ In Pakistan the government has ordered a 20% cut in school fees,^17^ amid lockdowns affecting the lower and middle class sadly the educational sector remains grossly neglected in terms of state sponsorship. Sensitive sectors of human development appear to be wrested away by lobbies with myopic vision instead of non-discriminatory research being encouraged in the virtual environment.

### Language

There are complex needs of language teachers using computer technology in addition to acquisition and constant improvement in their skills that are required, in addition native teachers may need to face variety of teaching and learning cultures.^18^ Indo-Pak subcontinent abounds in local languages and according to Ramani S, in an Indian study concluded that content in learners own language is essential in developing countries as far as online academics is concerned and has proposed use of Roman script for using students own language for communication online will count.^15^

### Training, control and support of staff & faculties

In the absence of Information Technology (IT) staff in schools, permanent school staff needs to be trained to maintain their IT systems.^15^ In an article, Dhilla SJ reviewed the online academic faculty development and reported that online academic teaching profoundly impacts the pedagogical practice of teachers. Faculty encounter a sense of vulnerability while navigating online.^19^ A local study revealed medical students to have negative perception as regards online education necessitating improvement by faculty and administrators.^20^

Also, Tannehill DB et al. concluded that an organization’s ability to implement standard of instructional design and practice would result in positive experience of students in online academics.^21^ The authors of the current study, who are also senior faculty members, now involved in online academics, faces similar experience. In an Indonesian study by Hasibuan and Santoso, developed an Administration System for supporting online academics, however the on ground success of the system is still to be examined.^22^

### Student Assessment & Examinations

In some regions including Pakistan, though online learning has been recommended,^23^ the closure of schools due to COVID-19 emergency has occurred at a critical time for evaluations and critical academic events cancelled. According to Burgess & Sievertsen, internal assessments in institutions are being given less importance during this COVID-19 emergency resulting in cancellations. Here it should also be kept in mind that students who are not given actual grades followed by being given predicted grades influence the labor market for students.^24^ Online assessments being opted by many universities in place of conventional examinations may be faced with ethical considerations accompanied by assessment errors. In a study by Alruwais N et al. reported that in addition to benefits of online assessment, it also faces barriers like poor technical infrastructure especially in developing countries; unfamiliarity of students with the hardware, software as well as assessment process; scoring issues; as well as difficulties in assessment of projects given to groups.^25^

### Online Security Issues

The platforms, soft wares, applications deployed for online academics are also liable to security breach. Layefa & Jackson in a Nigerian study concluded that such systems insecurity of authentication especially of the student are major challenges recommending that Nigerian institutions, should adopt security measures and ensure safety of transfer of data using encryption processes.^26^ It may be endeavored to video-audio record assessments to ensure transparency.

### Impact on Social Skills and Awareness

With loss of conventional face to face learning, social skills and social awareness, which are advantages of a conventional system may be affected in an online system. In a study by Carlsson M et al. to find impact of days of schooling on cognitive skills, it was reported that ten additional days of schooling raises results on intelligence tests including synonyms and technical comprehension by one percent of standard deviation with no additional impact of off school days.^27^ Also another study showed the positive effect of instructional time on test scores, however the effect was lower in developing countries and higher for schools with accountability measure.^28^ In context of Pakistan, lack of immediate student feedback in on-line lecturing, teachers faced difficulty assessing understanding of students and students misbehavior was also noted along with unethical access to online resources and for these acquiring of better software’s as well as those required for proctoring are suggested.^29^

### Mental Health

Mental health issues are on the cards during this COVID-19 lockdown,^30^ as the looming economic meltdown broods ill results for the outlook of vulnerable students and they require emotional counseling during these arduous times. Online academics may miss such issues.^8^ In a study Papadatou-Pastou M et al. reported online support systems created to support mental health, however only some cater to higher education learners^31^ impacting the mental health of students in this online academic era brought upon by COVID-19.

Policy makers need to take cognizance of rapidly evolving academic settings to ensure an uninterrupted standardized online teaching in this unforeseen situation, which according to Hashmi AM et al. has the capacity to bring changes in education sectors in countries like Pakistan^32^ and for sustainable reliability of online academics.

## CONCLUSIONS

Lockdown of centers of learning globally has caused significant distortion in the academic world but unequal interruption in learning with significant disruptions in internal assessments and qualification examinations with developing countries like Pakistan, compounded by a compromised educational system as evidenced by the penchant and preference for even dubious foreign degrees, makes all equally affected. However, it should be borne in mind that COVID-19 is spurring the case for conversion to online academics and developing countries like Pakistan are poised to develop reliable, cost effective and secure online academic system. Whether it is going to be a bane or boon can be considerably influenced by time, resources made available and concerted efforts made by the relevant stakeholders.

### Authors’ Contribution:

**Nazia Mumtaz:** Conceptualization of work, designing of research, Analysis & Interpretation & responsible for integrity of the work.

**Ghulam Saqulain:** Writing of Manuscript, Methodology, Literature Review & Finalization for publication.

**Nadia Mumtaz:** Critical revision of article.
